# Voices of the Pacific: Pacific Peoples’ Conceptualisations of Anxiety in Aotearoa New Zealand—Findings from a Cross-Sectional Survey

**DOI:** 10.3390/ijerph23030291

**Published:** 2026-02-26

**Authors:** Lorisha A. Chandra, Sarah McLean-Orsborn, Pesetā Veronica Tone-Graham, Sarah A. Kapeli

**Affiliations:** 1 School of Psychology, Faculty of Science, The University of Auckland, Auckland 1142, New Zealand; 2 School of Māori Studies and Pacific Studies, Faculty of Arts and Education, The University of Auckland, Auckland 1142, New Zealand

**Keywords:** anxiety, Pacific peoples, Pacific mental health, mental health literacy, psychological distress, help-seeking, New Zealand

## Abstract

**Highlights:**

**Public health relevance—How does this work relate to a public health issue?**

- Pacific peoples experience disproportionately higher rates of anxiety, alongside low engagement with formal mental health services.
- The study goes beyond prevalence research to explore how Pacific peoples understand and conceptualise anxiety.

**Public health significance—Why is this work of significance to public health?**

- Understanding how Pacific peoples conceptualise anxiety is essential for improving cultural congruence within mental health service provision.
- The findings challenge deficit-based assumptions about Pacific peoples’ mental health literacy by demonstrating their nuanced understandings of anxiety.

**Public health implications—What are the key implications or messages for practitioners, policy makers and/or researchers in public health?**

- Mental health services and policies must move beyond Western, symptom-based models and embed Pacific-led, culturally responsive approaches to mental health care.
- Pacific peoples’ underutilisation of mental health services is not necessarily due to a lack of knowledge, but indicative of structural and practical barriers to care.

**Abstract:**

Pacific peoples in Aotearoa New Zealand (Aotearoa is the Indigenous Te Reo Māori name of New Zealand) experience disproportionately higher rates of anxiety than the general population. However, while informal relational support is strongly utilised, formal or professional help-seeking remains comparatively low. Understanding how Pacific peoples conceptualise anxiety is critical for addressing this disparity. This study provides a snapshot of Pacific peoples’ understandings of anxiety in Aotearoa NZ. A total of 548 Pacific peoples aged 16 to 83 years who resided in Aotearoa NZ completed the Pasifika Mental Health in Aotearoa (PMHA) survey between 2018 and 2019, which included questions about anxiety. Inductive Content Analysis (CA) grounded by Pacific epistemologies was used to categorise open-ended responses, and participants’ response frequencies were analysed. The findings suggest that anxiety was understood as a transient, everyday experience, rather than a prolonged mental health condition. Informal relational support networks were strongly preferred in addressing or managing anxiety, followed by professional support. Perceived causes of anxiety were complex and evenly attributed to experiential, contextual, and health-related risk factors, highlighting the centrality of holism in Pacific worldviews. These findings suggest a nuanced understanding of anxiety that challenges deficit-based assumptions about Pacific peoples’ Mental Health Literacy (MHL), and emphasises the ongoing need for more culturally responsive, community-based, relational, and holistic mental health support.

## 1. Introduction

Anxiety disorders (commonly referred to as anxiety) are among the most prevalent mental health conditions worldwide, affecting 4.4% of the global population (approximately 359 million people) [1]. The Diagnostic and Statistical Manual of Mental Disorders, 5th Edition Text Revision (DSM-5-TR), characterises anxiety disorders as involving persistent and excessive future-oriented fear or worry that significantly disrupts behavioural, emotional, and physiological functioning [2]. These disruptions are often accompanied by symptoms such as restlessness, fatigue, irritability, muscle tension, and sleep disturbances [2]. The DSM-5-TR [2] further classifies anxiety disorders into distinct categories that could be experienced together, including Generalised Anxiety Disorder (GAD), Social Anxiety Disorder, Panic Disorder, Separation Anxiety Disorder, Agoraphobia, Specific Phobias, and Selective Mutism.

Aotearoa New Zealand (Aotearoa is the Indigenous Te Reo Māori name of New Zealand, hereafter referred to as Aotearoa NZ) records one of the highest global anxiety rates in the world (7537 cases per 100,000) [3]. Pacific peoples experience higher rates of anxiety (16.2%) in comparison to the general population (14.8%) and have greater comorbidities associated with anxiety [4,5,6]. While 8% of Pacific adults in Aotearoa NZ are diagnosed with a mood and/or anxiety disorder, only 25% of those diagnosed had sought support from mental health services, compared to 58% of the general population [4]. These disparities are driven by barriers to care that reduce timely access to mental health support [4,5,7,8]. Such barriers include stigma, discrimination, socioeconomic deprivation, reliance on family-based support, conflicting priorities, historical mistrust in formal support services, and limited knowledge or access to culturally grounded mental health support [7,8,9,10]. These barriers may be accompanied by practical challenges like cost or transport [4,5,7,8,9,10,11,12]. Despite these ongoing disparities, existing research on Pacific conceptualisations of anxiety (and mental health more broadly) has predominantly focused on prevalence, service utilisation, and structural barriers to care. Research on how anxiety is understood or conceptualised within Pacific communities is limited. Large-scale national surveys, such as Te Rau Hinengaro: The New Zealand Mental Health Survey [4], have documented rates of anxiety disorders among Pacific peoples, while more recent reports, including Te Kaveinga [13], have highlighted service access issues in Aotearoa NZ. However, a key gap across these existing studies is that they do not examine how Pacific peoples themselves define, interpret, or experience anxiety. Consequently, there remains limited empirical evidence exploring Pacific conceptualisations of anxiety, their help-seeking preferences, and perceptions of what causes anxiety [7,14].

Mainstream Western models of mental health are grounded in individualism and diagnostic categorisation that often fail to account for the relational, spiritual, and collective dimensions of how wellbeing is understood and expressed across Pacific worldviews [15,16,17]. Instead, Western frameworks classify anxiety as a discrete clinical disorder based on neurochemical and cognitive dysfunction, with treatment through psychotherapeutic or pharmacological interventions [18,19]. Meanwhile, Pacific worldviews locate wellbeing within relational harmony, where distress is often expected to be managed collectively through existing connections within family, church, or community contexts [20,21,22]. The concept of Vā captures this relational ontology, referring to the sacred, unseen, relational spaces that connect people to one another and to places [23,24]. Furthermore, Mental Health Literacy (MHL) refers to the knowledge and beliefs that facilitate the recognition, management, and prevention of mental health challenges [25]. Yet, Pacific peoples’ understandings of MHL are shaped by cultural worldviews, relational values, and collective experiences of health and illness [14]. Dominant clinical frameworks often lack this cultural alignment, where terms such as “anxiety” may not translate meaningfully into Pacific contexts and, instead, contribute to the under-recognition of distress and delayed engagement with support services [17,26].

These collectivist and relational understandings of wellbeing are evident across many cultural contexts globally and differ from Western biomedical frameworks. More specifically, in the Pacific, the Fonofale Model [27] is a foundational example of how Pacific wellbeing is holistic and relational, emphasising the inseparability of mental health from core Pacific values [15,20]. Represented as a *fale* (Sāmoan house), the model conceptualises wellbeing as the interconnected balance of all types of wellbeing (social, physical, mental, and environmental). The model positions family as the foundation, with cultural values and beliefs forming a circular-shaped roof, supported by spiritual, physical, and mental wellbeing as pillars that uphold the structure, and are shaped by time, environment, and context. Although not a diagnostic tool, the Fonofale model provides a culturally grounded guide for understanding distress as a disruption of balance across these relational domains rather than as an isolated psychological pathology [27]. Moreover, prior research in the area has employed standardised assessment tools and guides, such as the DSM-5-TR [2], which may increase the risk of missing cultural nuances due to their largely clinical, symptom-based approach. Hence, despite the higher rates of anxiety within Pacific communities, the underutilisation of mental health support by Pacific peoples in Aotearoa NZ may be indicative of these ongoing cultural misalignments within mental health support. Addressing this gap requires further research and an understanding of how Pacific peoples conceptualise mental health and anxiety.

The present study provides a snapshot of how anxiety is understood by Pacific peoples in Aotearoa NZ. A three-part focus has been employed to (1) conceptualise what anxiety is, (2) identify preferences to best manage anxiety, and (3) identify perceptions of what risk factors may cause anxiety. This study is situated within Pacific research paradigms that emphasise relational accountability, cultural grounding, service, and responsibilities to our communities. Hence, it is important to clarify the epistemological positioning informing this study. The research team comprises Pacific researchers with ethnic links to Fiji (Chandra), Tonga (Kapeli), and Sāmoa (McLean-Orsborn & Tone-Graham), allowing this research to be grounded in Pacific ways of knowing and being. As such, this research contributes to growing evidence on Pacific understandings of mental health that may be drawn upon to inform culturally grounded mental health support for Pacific communities in Aotearoa NZ.

## 2. Methods

### 2.1. Researcher Positioning

This study was undertaken by a team of Pacific mental health researchers whose cultural and genealogical ties to Pacific communities inform the epistemological grounding of the methodology. In Pacific research, knowledge is relational, contextual, and shaped by the researchers’ values. Accordingly, researcher positionality is not peripheral, but integral to methodological transparency as it informs the analytical approach and interpretation of the findings [28]. Specifically, this positioning ensures the findings remain grounded in Pacific lived realities and are reflective of their cultural context. Hence, positionality is presented here as a source of accountability and reflexivity that shapes the overall research, and the researchers who are embedded in it. This approach ensures that Pacific research is done with, by, and for Pacific peoples.

### 2.2. Project Overview

The present study draws upon data from the Pasifika Mental Health in Aotearoa (PMHA) project led by Dr. Sarah Kapeli at the University of Auckland in Aotearoa NZ [10,14]. The PMHA was developed as part of a larger project exploring Pacific MHL, including the PMHA survey that is used in the current study. This cross-sectional survey (online and paper version) collected information from 2018 to 2019 from 548 self-identified Pacific peoples. Survey eligibility criteria included being 16 years of age or older, identifying with a Pacific ethnicity, and normally living in Aotearoa NZ. The survey was initially developed by drawing upon personal knowledge, existing resources, the literature, and surveys pertaining to Pacific mental health and mental health more broadly. Once an initial survey draft was compiled, a Talanoa (multi-Pacific practice involving the sharing of ideas, stories, and aspirations through conversation) was organised with the PMHA’s Pacific Advisory Team (PAT), which included: Professor Jemaima Tiatia-Siau, Dr. Sione Vaka, Dr. Epenesa Olo-Whaanga, Dr. Sam Manuela and Mrs. Sisilia Noavea. The PAT provided cultural, clinical, community, and academic expertise to further refine the survey.

The survey was then piloted with volunteers from Pacific communities, whose feedback was used to further refine it. After a final consultation with the PAT, the PMHA survey was complete. Survey participation was opt-in and entirely voluntary. Recruitment involved a combination of direct and snowball sampling methods. A unique follow-on effect of direct sampling is snowball sampling (also known as chain-referral sampling), in which participants identify and invite other potential participants. This method is particularly effective for exploring sensitive topics and increasing participation through Pacific peoples’ existing relational networks [19,29,30]. To enhance recruitment, advertisements were displayed in public spaces and on social media platforms such as Facebook and Instagram.

### 2.3. Ethics

The University of Auckland Human Participants Ethics Committee (UAHPEC) approved the survey and supporting documents from 29 October 2018 until 29 October 2021. Reference number: 022137. In Aotearoa NZ, the legal age for informed consent to participate in research is 16 years; therefore, individuals aged 16 years and older were eligible to provide consent independently [31]. The survey could be completed online via Qualitrics or as a physical paper copy. Participants were informed that the survey was voluntary, anonymous, and confidential via a Participant Information Sheet (PIS). As outlined in the PIS, the voluntary submission of the survey constituted informed consent.

### 2.4. Participants

The survey included responses (99% completed online via Qualtrics) from 548 Pacific peoples (14.6% men, 84.8% women, 0.6% gender diverse) with an age range from 16 to 83 years (Median = 25; Mean = 27; and Standard Deviation = 9.98). Participants represented many Pacific nations; in many cases, identifying with more than one Pacific ethnicity. Of the four largest represented Pacific ethnic groups in Aotearoa NZ according to the latest Census [32], the survey responses included 291 participants who identified as Sāmoan (53.1% of survey sample versus 49.3% of Pacific population), 156 participants who identified as Tongan (28.5% of survey sample versus 20% of Pacific population), 62 participants who identified as Cook Island Māori (11.3% versus 19.1% of Pacific population), and 62 participants who identified as Niuean (11.3% versus 8.1% of Pacific population) (see Table 1).

### 2.5. Survey Description

Data is drawn from the PMHA survey, a cross-sectional survey (online and paper version) that collected information from 2018 to 2019 from 548 self-identified Pacific peoples aged 16 years and older who normally live in Aotearoa NZ. The survey comprised seven sections related to socio-demographics, personal experiences with mental health, personal attitudes towards mental health, personal knowledge about mental health promotion activities, knowledge about depression, and knowledge about anxiety. The principles of Talanoa were drawn upon in the survey design to ensure culturally safe, accessible, and authentic ways of representing Pacific voices. Talanoa is underpinned by key Pacific values, including ‘ofa (love, care, and kindness), māfana (warmth), mālie (humour and connection), and tauhi vā (nurturing and sustaining connections), which together guided the structure and delivery of the survey.

For instance, open-ended questions were prioritised in the survey to enable self-disclosure and deeper thought than what tick-box responses allow. Anonymity was important to ensure participants felt safe and confident. Many survey sessions were held during the data collection phase to complete the survey (with or without support), ask questions, and Talanoa (sharing ideas and stories through conversation). These sessions provided time and space alongside food, music, laughter, and sometimes tears between participants and researchers to foster a safe, warm, and welcoming environment. Paper copies were also available to make the survey more accessible. These design choices ensured that the survey was more than just a data collection tool; it was a process reflective of Pacific values.

### 2.6. Survey Questions

The present study focuses on three PMHA survey questions related to anxiety. For all items, participants were presented with the question and could provide an open-ended response. For ‘Item 1’, Participants were presented with the question, ‘What do you think ‘anxiety’ is?’ For ‘Item 2’, Participants were presented with the question, ‘If you or someone you know has anxiety, what do you think is the best thing to do?’ For ‘Item 3’, Participants were presented with the question, ‘What do you think are the causes of anxiety?’

### 2.7. Analytical Approach

The present study is a mixed qualitative-quantitative study that primarily focuses on participants’ open-ended responses to the PMHA survey questions about anxiety. The data was analysed, and participant responses were coded by the first author using Inductive Content Analysis (CA). CA is a systematic approach for identifying and analysing patterns in qualitative and quantitative research, and is widely used in healthcare, social and community psychological research [33,34,35,36]. CA quantifies the occurrence of concepts, patterns, or keywords within a dataset. Often, a deductive approach is used, utilising predefined codes to objectively describe surface-level characteristics in a standardised way. Alternatively, the present study employs an inductive approach, allowing patterns and key ideas to be derived from the responses [33,34,35,36]. CA also allowed for new insights to surface without being constrained by existing frameworks, providing a culturally responsive method for working with Pacific data.

While CA also focuses on summarising and quantifying data, findings are often presented in numerical and statistical formats or through textual explanations and interpretations, as in the present study. The CA framework aligned with the study’s aim to privilege Pacific voices, ensuring that the findings remained culturally grounded and robust. To enhance rigour and minimise bias, coding definitions were developed iteratively through repeated engagement with the data, and regular review from the co-authors. The resulting coded categories were refined collaboratively to ensure clarity and internal consistency, rather than being analysed in parallel. Any coding discrepancies were resolved by reaching consensus. These final codes were quantified in statistical software (SPSS Version 30) and presented descriptively as participants’ response frequencies.

## 3. Results

### 3.1. Question 1

The CA of participant responses to the question ‘What do you think ‘anxiety’ is?’ yielded 10 categories. Table 2 presents these categories, their definitions, and the frequency with which they appeared in participant responses. Of the 548 survey participants, 427 participants responded to this question. Given that each response could be coded across multiple categories, the analysis resulted in 1478 coded segments.

The most frequent category was ‘Identified general symptoms of anxiety’ (*n* = 773; 52%), with participants describing common experiences, such as ‘worry,’ ‘overthinking,’ or ‘fear’, whereas fewer participants identified symptoms according to the DSM-5-TR criteria (*n* = 177; 12%). A smaller proportion of participants recognised anxiety as an illness (*n* = 51; 3%) or as a condition characterised by persistent symptoms (*n* = 34; 2%). Despite the awareness of symptoms, participants did not necessarily identify when these experiences became indicative of a mental health condition. The second most frequent category, ‘Experiencing multiple symptoms attributable to anxiety’ (*n* = 365; 25%), reflected awareness of anxiety as an experience that may affect thoughts, emotions, and behaviours. Together, these findings suggest that Pacific peoples widely recognise anxiety symptoms and tend to conceptualise anxiety as an everyday, transient experience. Limited alignment with DSM-based frameworks should not be interpreted as poor MHL. Instead, it may reflect different ways of understanding wellbeing, recognising that everyday expressions of anxiety may not always mirror formal diagnostic terms.

### 3.2. Question 2

The CA of participant responses to the question ‘If you or someone you know has anxiety, what do you think is the best thing to do?’ yielded 7 categories. Table 3 presents these categories, their definitions, and the frequency with which they appear in participant responses. Of the 548 survey participants, 411 participants responded to this question. Given that each response could be coded across multiple categories, the analysis resulted in 890 coded segments.

The most frequent category identified was ‘Seek Informal Support’ (*n* = 524; 59%), highlighting the central role of relational connectedness in addressing anxiety within Pacific contexts. The second most frequent category was ‘Seek Formal Support’ (*n* = 127; 14%), indicating recognition of anxiety as a condition that may require professional care. These findings suggest an awareness of anxiety as a health condition requiring some specialised or professional care, and that while Pacific peoples value seeking support, the preference for support through existing relational networks, such as family and friends, is much stronger. Participants also identified individual strategies to manage anxiety, including self-education or self-care (*n* = 61, 7%) and physical activities (*n* = 104, 12%), reflecting awareness of the mind–body connection. Overall, these findings suggest that Pacific peoples value help-seeking, contrary to deficit-based assumptions about Pacific peoples’ MHL and help-seeking behaviours. Furthermore, they indicate a need for relational, community-based support measures that could enhance existing formal support.

### 3.3. Question 3

The CA of participant responses to the question ‘What do you think are the causes of anxiety?’ yielded 4 categories. Table 4 presents these categories, their definitions, and the frequency with which they appear in participant responses. Of the 548 survey participants, 402 participants responded to this question. Given that each response could be coded across multiple categories, the analysis resulted in 898 coded segments.

Participants identified a broad and evenly distributed range of perceived causes, indicating that the risk factors causing anxiety are complex. The most frequent category was ‘Caused by environmental and/or situational pressures’ (*n* = 328; 37%), reflecting perceptions that anxiety arises from external stressors and structural demands, rather than individual pathology. The second most frequent category, ‘Caused by experiential factors’ (*n* = 251; 28%), linked anxiety to life events and lived experiences, highlighting a temporal and relational understanding of anxiety. Similarly, 255 participants (28%) identified health-related factors as perceived causes of anxiety, including references to physical health and mental wellbeing. Together, these findings are consistent with holistic and relational models of wellbeing worldwide and are pertinent to Pacific communities (see The Fonofale Model) [27]. Such multidimensional understandings of wellbeing recognise that many interconnected factors shape experiences of anxiety, and wellbeing overall [27].

## 4. Discussion

The present study addressed its stated objectives. Specifically, the analysis enabled insights into (1) the conceptualisation of how anxiety is understood by Pacific peoples in Aotearoa NZ, (2) the identification of preferences for managing anxiety, and (3) the identification of perceived risk factors that cause anxiety. Although these findings may not represent the wider Pacific population in Aotearoa NZ, they successfully provided a snapshot of how anxiety is understood within the present sample.

The findings indicate that Pacific peoples in Aotearoa NZ have a strong understanding of anxiety, most often conceptualising it as an everyday, transient experience. Participants identified a broad range of symptoms that aligned with DSM-5-TR diagnostic descriptions of anxiety. However, only a small number of responses described anxiety as persistent or chronic in duration. According to the APA [2], chronicity is central to DSM-5-TR definitions of anxiety disorders, rendering anxiety as debilitating and problematic. The relative absence of this emphasis in participant responses highlights a divergence between diagnostic criteria and how anxiety was described within the present sample. The findings also showed a strong preference for informal forms of support, with participants most often turning to family, friends, and community networks when experiencing anxiety. This preference was almost four times greater than preferences for formal support options (e.g., doctors, mental health specialists), and reflects the centrality of relationality and Vā within Pacific communities [23,37,38]. Consistent with Pacific frameworks [39,40], such as the Fonofale Model [27] and prior research [10,22] that has indicated the centrality of social connections for Pacific peoples, wellbeing is grounded in family and collective relationships. As such, Pacific communities often understand and manage distress communally, rather than individually [14,37]. Additionally, participants did not reject formal mental health services, but viewed them as complementary to informal support. These findings align with previous literature [7,8,13,14,41] highlighting practical and structural barriers that may shape formal service utilisation.

Understanding the perceived causes of anxiety is critical, as they shape how distress is interpreted and managed within Pacific communities. Findings revealed an almost equal distribution of perceived causes across environmental, experiential, and health-related factors, including academic or work pressures, past trauma, and existing health conditions. This distribution indicates that participants identified multiple domains in which anxiety may arise, rather than locating causes within a single category. While participants’ responses were coded individually and do not imply that each person endorsed all domains, the overall pattern is consistent with holistic understandings of wellbeing. Such worldviews recognise that anxiety may arise from interconnected social, physical, relational, and environmental influences, rather than as a discrete psychological condition. While holistic perspectives are evident across diverse cultural contexts, Pacific understandings [27,39,40] are unique in their view of distress as a disruption of balance across these dynamic connections. This is evident in the Fonofale Model [27] and Kapeli [14], where the model symbolises the wholeness of a Pacific person, drawing upon values of the broader Pacific community such as family, culture, and spirituality (refer to Talanoa values in Survey Description). Health-related explanations further emphasised the inseparability of the mind and body, with physical sensations (e.g., heart palpitations, restlessness, fatigue) linked to anxiety. Together, these findings suggest that clinical practices may benefit from holistic assessment and treatment pathways that align with Pacific worldviews.

The findings from Question 2 provide a clear basis for shaping culturally aligned support pathways. With 59% of participants endorsing ‘Seeking Informal Support’, strengthening interventions that operate within existing relational networks may be particularly relevant. Rather than positioning formal services as separate from these networks, integrating care within familiar Pacific environments (churches, sports clubs, and community centres) may enhance accessibility, trust, and engagement [42]. The identification of formal support (14%) further suggests that professional services are recognised as important but may be most effective when delivered in a complementary way. Collaborative approaches such as family-centred therapy [43] and Talanoa-based talking therapies [44,45,46] can complement formal interventions, including Cognitive Behavioural Therapy (CBT) [19], and position Pacific peoples and their families as knowledge holders. Partnerships with community leaders, clinicians, Pacific advisors, councils, and mental health advocates may further support funding, psychoeducation, and outreach initiatives that address structural determinants of wellbeing and provide guidance on working with Pacific clients [8,42]. Notably, 28% of responses to Question 3 identified health-related factors as a perceived cause of anxiety. This emphasis on the mind–body connection aligns with Wendt [37], who suggests that embodied forms of support may resonate with Pacific peoples’ experiences of anxiety. As Whistler [21] suggests, this may look like incorporating and normalising traditional Pacific healing practices. These proposals emerge directly from the observed results, suggesting that formal services may be strengthened through relational integration and cultural alignment.

### Strengths and Limitations

A key strength of this study is its non-invasive, culturally grounded design, which centres Pacific voices rather than imposing external Western frameworks. Guided by Talanoa principles, the use of open-ended survey questions enabled participants to express their understandings in their own words. Inductive content analysis strengthened methodological rigour by minimising researcher-imposed biases and capturing diverse responses. The study also examined multiple interconnected dimensions of anxiety, and the survey was collaboratively designed with a Pacific advisory group. The diverse, large sample (*n* = 548) represented a range of demographics, including Pacific ethnic groups, age cohorts, and qualifications, offering perspectives on anxiety and mental health that are seldom captured in mainstream research.

Despite its strengths, several limitations must be considered. The study did not disaggregate findings by specific Pacific ethnic groups (e.g., Sāmoan, Tongan, and Cook Island Māori), limiting insight into culturally distinct conceptualisations of anxiety. Future research should explore variations in how anxiety is conceptualised across specific Pacific age groups, by gender, and ethnic group, as these demographic differences may shape experiences and help-seeking patterns. Given this, the sample was not fully representative of the wider Pacific population, with an overrepresentation of younger participants (<45 years), females, and Auckland-based participants. This demographic bias limits generalisability to older adults, Pacific men, and those residing outside of urban regions in Aotearoa NZ. The online, English-language survey design may have introduced self-selection and language biases, excluding individuals with limited digital access or English proficiency. Methodologically, the cross-sectional design of this study provides only a snapshot in time, preventing an examination or comparison of how conceptualisations of anxiety may shift across generations, migration histories, policy changes, or global events. Reliance on survey data limited the depth of insight achievable through Talanoa-based qualitative methods. Future research should consider longitudinal approaches to explore conceptualisations of anxiety over time and enable richer cross comparisons. Moreover, future studies should examine how these findings can be translated into practice.

## 5. Conclusions

The present study centres Pacific voices, providing insight into how Pacific peoples in Aotearoa NZ understand, experience, and respond to anxiety disorders. The findings reveal a strong understanding of anxiety as an everyday, transient experience, rather than a persistent or prolonged condition, alongside a strong preference for informal, relational support measures to best manage and address anxiety, complemented by professional services. Additionally, Pacific peoples attribute the perceived causes of anxiety to various experiential, contextual, and health-related pressures. These insights challenge deficit-based assumptions about MHL in Pacific communities, highlighting the need for community-based, culturally responsive mental health approaches that recognise and meaningfully integrate Pacific values, needs, and lived experiences. Moreover, the findings reiterate that effective mental health support is never an individual pursuit, but a collective responsibility towards sustained wellbeing.

## Figures and Tables

**Table 1:** Participant demographic information.

		% Yes
Gender	Men	14.6
	Women	84.8
	Gender Diverse	0.6
Pacific Ethnicity		
	Sāmoan	53.1
	Tongan	28.5
	Cook Island Māori	11.3
	Niuean	11.3
	Fijian	6.4
	Tokelauan	2.6
	Tuvaluan	0.9
	Tahitian	0.2
	Kiribati	0.5
	Papua New Guinean	0.5
	Solomon Islanders	0.4
	Rotuman	0.4
	Hawaiian	0.2
	Pitcairn Islander	0.2
	Also identified as New Zealand Māori	13.3
	Also identified with non-Pacific ethnicity	24.5
Age Group		
	16–24	48.4
	25–44	45.1
	45–64	5.8
	65+	0.8
Location based on regional council		
	Auckland	73.6
	Wellington	10.1
	Canterbury	3.6
	Waikato	3.4
	Bay of Plenty	2.7
	Manawatu-Wanganui	2.3
	Otago	2.3

Note. *n* = 548.

**Table 2:** Participant response categories and response frequency for the question ‘What do you think anxiety is?’.

Category	Definition	Response Frequency *n =* 427 (Cumulative % of Total)
Do not know	Responses indicate a lack of understanding or knowledge about the concept of anxiety. This category encompasses statements such as ‘I don’t know’, ‘I’m not sure’, or any response that demonstrates an inability to define or describe anxiety.	19 (1)
Recognise as an illness	Responses that explicitly identify anxiety as a recognised illness. This category includes statements that refer to anxiety as a ‘disorder’, ‘disease’, ‘illness’, ‘mental health condition’, ‘mental health state’, or similar terminology without specifying symptoms or duration.	51 (3)
Persistent symptoms over time	Responses that highlight the characteristics of anxiety as involving symptoms that persist over an extended period. This category includes statements that mention symptoms lasting for ‘week’, ‘a long time’, ‘all the time’, ‘chronic’, or any indication of a prolonged duration.	34 (2)
Experiencing multiple symptoms attributable to anxiety	Responses that list or describe multiple symptoms (two or more commonly associated with anxiety).	365 (25)
Identified symptoms of anxiety according to the DSM-5-TR	Responses that explicitly list or describe symptoms of anxiety that align with the diagnostic criteria outlined in the DSM-5. This category requires an accurate and specific mention of symptoms, such as ‘excessive anxiety and worry’, ‘difficulty feeling in control’, ‘significant restlessness or feeling on-edge’, ‘fatigue’, ‘difficulty concentrating or mind blank’, ‘heightened irritability or mood swings’, ‘significant muscle tension’, ‘insomnia or hypersomnia’.	177 (12)
Identified symptoms that are not attributable to anxiety	Responses that list or describe symptoms that are not typically associated with anxiety, or that are more indicative of other conditions. This category includes symptoms that are not considered core features of anxiety according to established diagnostic criteria, or symptoms that may be better explained by other medical or psychological conditions and included ‘diminished interest’, ‘feelings of dread’, ‘hearing a critical inner voice’.	8 (1)
Identified general symptoms of anxiety	Responses that list or describe symptoms commonly associated with anxiety, but without necessarily adhering to the specific criteria of the DSM-5. This category includes general mentions of symptoms like feeling worried, overwhelmed or overthinking, negative thoughts, feeling scared or afraid, nervousness, paranoia, anger, stress, isolation, discomfort, insecure, increased heart rate, sweating, breathing issues, panicking, nausea, or fatigue, without requiring detailed or specific descriptions.	773 (52)
Response is outside the scope of the question	Responses that were outside the scope of the questions, were unrelated to the question, indiscernible, or were related to the causes of anxiety.	27 (2)
Disrupts Relationships	Responses that explicitly mention the impact of anxiety on relationships with individuals (e.g., family members, friends, partners), specific places (e.g., faith-based place, work, school, university), or another space (e.g., social gatherings, public events). This category requires the identification of a particular relationship, place, or space that is negatively affected by anxiety.	10 (1)
Described Metaphorically	Responses that use figurative language, metaphors, or analogies to describe anxiety. This category includes statements that use symbolic or imaginative language to convey the experience of anxiety rather than literal descriptions of symptoms.	14 (1)

**Table 3:** Participant response categories and response frequency for the question ‘If you or someone you know has anxiety, what do you think is the best thing to do?’.

Category	Definition	Response Frequency *n =* 411 (Cumulative % of Total)
Do not know	This category captures responses indicating uncertainty about how to respond to anxiety, encompassing statements such as ‘I don’t know’ or ‘I’m not sure’.	38 (4)
Seek Formal Support	This category encompasses responses suggesting that anxiety requires assistance from formally trained healthcare providers or mental health specialists. This category includes responses that indicated ‘see the Doctor’, ‘seek medical help’, ‘talk to a counsellor’ or ‘get a referral to a psychologist’.	127 (14)
Seek Informal Support	This category refers to responses suggesting that individuals with anxiety should turn to their existing social networks for help and comfort. This category includes responses that indicated that ‘talking to family members’, ‘talking to friends’, ‘spending time with others’, ‘seeking emotional support from others’, ‘breathing exercises’, ‘mindfulness or meditation’, and ‘knowing your coping strategies’.	524 (59)
Seek Support in General	This category refers to responses suggesting that individuals with anxiety should turn to some form of support, without anything explicitly mentioned in detail.	25 (3)
Engage in Spiritual Support	This category refers to responses suggesting that individuals with anxiety should turn to spiritual support for help, direction, and comfort. This category includes responses that indicated ‘connect with God or a higher being’, ‘talk to God or praying’, ‘attend religious groups and activities’ and ‘talk to spiritual leaders.’	11 (1)
Engage in Personal Strategies	This category includes responses suggesting that individuals can take personal actions to manage or alleviate their anxiety symptoms. This category includes responses that indicated ‘learn psychotherapeutic techniques’, ‘self-education’, ‘taking space’, ‘self-care’, ‘crying’, ‘engaging in a mental distraction.’	61 (7)
Engage in Physical Strategies	This category includes responses suggesting that individuals can take physical actions to manage or alleviate their anxiety symptoms. This category includes responses that indicated ‘exercising’, ‘resting or sleeping’, ‘being in nature’, ‘being in a safe physical space’, ‘seeking physical comfort’, ‘engage in activities’, and ‘listening to music’.	104 (12)

**Table 4:** Participant response categories and response frequency for the question ‘What do you think are the causes of anxiety?’.

Category	Definition	Response Frequency *n =* 402 (Cumulative % of Total)
Do not know	This category captures responses indicating uncertainty about the causes of anxiety, encompassing statements such as ‘I don’t know’ or ‘I’m not sure’.	64 (7)
Caused by environmental and/or situational pressures	This category captures responses indicating that external or situational pressures and stressors are the primary cause of anxiety. This category encompassed statements of ‘high workload’, ‘meeting deadlines’, ‘academic pressure’, ‘family expectations’, ‘intergenerational trauma’, ‘lack of support’, ‘family genetics’, ‘parenting or upbringing’, ‘relationship conflict’, ‘bullying’, ‘fear of social situations and interactions’, ‘fear of crowds’, ‘financial uncertainty’, ‘housing insecurity’, ‘poverty’, ‘feeling unsafe’, ‘socioeconomic stressors’, ‘political and historical factors’, ‘social media and media’, ‘fear of failure’, ‘social comparison’, ‘societal expectations’, ‘perfectionism’, ‘lack of control’, ‘identity loss’, and ‘fear or worry’. It also included general mentions that anxiety is caused by our environment or situations, without explicit detail.	328 (37)
Caused by experiential factors	This category captures responses indicating that anxiety is caused by significant events or life experiences. It encompasses statements of ‘past events or traumas’, ‘historical abuse’, ‘stressful and negative experiences’, ‘childhood trauma’, ‘unpredictable situations’, ‘influence of technology’, ‘stress about the future’, ‘grief’, ‘identity loss’, ‘major life changes’, and ‘high responsibilities.’	251 (28)
Caused by health-related factors	This category captures responses indicating that anxiety is caused by health-related factors, including physical and mental and emotional wellbeing. It encompasses statements including ‘poor physical health’, ‘lack of sleep’, ‘existing health issues’, ‘chemical and biological causes’, ‘poor diet’, ‘using drugs and alcohol’, ‘low self-esteem’, ‘burnout and fatigue’, ‘innate sense of worry’, ‘maladaptive thought patterns’, ‘catastrophising and paranoia’, ‘mood swings’, ‘dysregulation’, ‘personality traits’, ‘other mental health issues’ such as ‘PTSD’ or ‘OCD’, ‘intense stress’, ‘depression’ and ‘feeling in flight or fight mode’.	255 (28)

## Data Availability

The original contributions presented in this study are included in the article. Further inquiries can be directed to the corresponding author.

## Abbreviations

The following abbreviations are used in this manuscript:

MHL	Mental Health Literacy
DSM-5-TR	Diagnostic and Statistical Manual of Mental Disorders 5th Edition Text Revision
